# Impact of the 2019 Food and Drug Administration Guidance for Uncomplicated Urinary Tract Infection on Treatment Response Rates: A Reanalysis of a Clinical Trial of Nitrofurantoin vs Fosfomycin

**DOI:** 10.1093/ofid/ofad557

**Published:** 2023-11-04

**Authors:** Rebecca Grant, Andrea Büchler, William Flight, Chun Huang, Diogo Ferrinho, Salim Janmohamed, Aruni Mulgirigama, Maciek Godycki-Cwirko, Leonard Leibovici, Angela Huttner, Stephan Harbarth

**Affiliations:** Infection Control Programme, Geneva University Hospitals and Faculty of Medicine, University of Geneva, Geneva, Switzerland; Infection Control Programme, Geneva University Hospitals and Faculty of Medicine, University of Geneva, Geneva, Switzerland; Clinical Sciences, GSK, Brentford, United Kingdom; Biostatistics, GSK, Collegeville, Pennsylvania, USA; Global Medical Affairs, GSK, Brentford, United Kingdom; Clinical Sciences, GSK, Brentford, United Kingdom; Global Medical Affairs, GSK, Brentford, United Kingdom; Centre for Family and Community Medicine, Faculty of Medical Sciences, Medical University of Lodz, Lodz, Poland; Rabin Medical Center, Beilinson Hospital, Petah Tikva, Israel; Faculty of Medicine, Tel-Aviv University, Ramat Aviv, Israel; Center for Clinical Research, Geneva University Hospitals and Faculty of Medicine, University of Geneva, Geneva, Switzerland; Division of Infectious Diseases, Geneva University Hospitals and Faculty of Medicine, University of Geneva, Geneva, Switzerland; Infection Control Programme, Geneva University Hospitals and Faculty of Medicine, University of Geneva, Geneva, Switzerland; Division of Infectious Diseases, Geneva University Hospitals and Faculty of Medicine, University of Geneva, Geneva, Switzerland

**Keywords:** acute cystitis, clinical trials, fosfomycin, nitrofurantoin, urinary tract infection

## Abstract

**Background:**

Current US Food and Administration (FDA) guidance recommends that the primary efficacy endpoint for uncomplicated urinary tract infection (uUTI) clinical trials be a composite of clinical and microbiological responses. We applied these criteria to a previous clinical trial to determine the impact on treatment outcomes.

**Methods:**

We conducted a patient-level reanalysis of a randomized clinical trial of nitrofurantoin versus fosfomycin for treatment of uUTI in nonpregnant adult women. Women were included in the reanalysis if they had 2 or more signs/symptoms of uUTI and a single bacterial species isolated from baseline urine culture at ≥10^5 ^colony-forming units (CFU)/mL. The applied primary efficacy endpoint—therapeutic response—required both clinical resolution of signs/symptoms and reduction of the infecting bacterial pathogen to <10^3 ^CFU/mL at day 14 post–treatment completion.

**Results:**

Two hundred eleven of 513 (41%) patients were eligible for inclusion in the reanalysis. Among these patients, 74% (76/103) and 69% (75/108) in the nitrofurantoin and fosfomycin groups, respectively, achieved clinical resolution by day 14. Similarly, 70% (72/103) and 67% (72/108) in each group achieved microbiological success at day 14. As such, 59% (61/103) and 57% (62/108) of women in each group met the primary efficacy endpoint—therapeutic success—at day 14. In comparison, 75% and 66% of patients in each group achieved clinical resolution at day 14 in the initial clinical trial.

**Conclusions:**

Applying current FDA guidance resulted in lower composite efficacy rates than clinical resolution alone as observed in the initial clinical trial. This may limit the ability to compare antibiotic treatment effects between historical and future clinical trials.

## Teaser

In a reanalysis of a randomized clinical trial of nitrofurantoin versus fosfomycin for treatment of uncomplicated UTI, applying current FDA guidance, we found efficacy as measured by composite therapeutic success rates to be lower than that defined by clinical resolution alone as observed in the initial clinical trial.

Uncomplicated urinary tract infection (uUTI) is a common bacterial infection for which nitrofurantoin and fosfomycin are among the recommended first-line therapeutics [1]. Given the prevalence of uUTI and increasing antimicrobial resistance [2–4], there is a need to develop novel therapeutic agents.

In August 2019, the United States Food and Drug Administration (FDA) published updated guidance for industry on studies evaluating therapeutics for the treatment of uUTI [5]. This introduced more stringent inclusion and efficacy endpoint criteria than those typically used in historical clinical trials for the treatment of uUTI, including previous registrational and nonregistrational trials, and previous regulatory guidance [6]. Specifically, the guidance requires women to have at least 2 of the following uUTI symptoms at baseline: dysuria, urinary frequency, urinary urgency, or suprapubic pain. The guidance also proposes that the identification of bacteria (single species of bacteria on pure culture recommended) from urine culture at ≥10^5^ colony-forming units per milliliter (CFU/mL) is considered a true bacterial pathogen [5]. In addition, the recommended primary efficacy endpoint is a composite endpoint of clinical and microbiological responses, assessed at a fixed timepoint after randomization based on the duration of treatment course and the half-life of the drug. This composite primary endpoint did not feature in the previous version of FDA guidance [6] and requires both clinical success, defined as complete resolution of all uUTI signs/symptoms and no new uUTI signs/symptoms; and microbiological success, defined as a reduction in the infecting bacterial pathogen to <10^3^ CFU/mL in urine culture. Updated guidance from the European Medicines Agency (EMA) also recommends a similar composite efficacy endpoint for the evaluation of therapeutics for uUTI [7].

In 2018, Huttner et al published the results of an investigator-initiated, multinational, open-label, analyst-blinded, randomized clinical trial of nitrofurantoin versus fosfomycin for the treatment of uUTI in nonpregnant women [8]. Among 513 nonpregnant women aged ≥18 years with uUTI who were randomized to receive 1 of the 2 study therapeutics, clinical resolution at day 28 post–treatment completion (ie, complete resolution of signs and symptoms of uUTI at days 14 and 28 post–treatment completion) was achieved in 70% of patients in the nitrofurantoin group and 58% of patients in the fosfomycin group, with the 12% (95% confidence interval, 4%–21%; *P* = .004) difference in efficacy between the 2 groups being statistically significant [8].

Since the publication of both the update to FDA guidance and the clinical trial by Huttner et al, no new antibiotics for the treatment of uUTI have been approved by regulatory authorities. Furthermore, the impact of these criteria on historical clinical trials for uUTI and how composite treatment responses may compare to other, less stringent efficacy endpoints remain unclear. The systematic collection of both clinical and microbiological data at baseline and day 14 and day 28 post–treatment completion lends the initial clinical trial cohort [8] to an application of the definition of therapeutic response based on updated FDA guidance to address this question. For this reason, we conducted a retrospective, patient-level reanalysis of the data from the initial clinical trial adapting the analysis population and endpoint definitions to reflect the 2019 FDA uUTI guidance.

The objective of the reanalysis was to determine the impact of the 2019 FDA uUTI guidance on treatment outcomes. Given the stringent regulatory guidance, we hypothesized that the proportion of patients meeting the composite endpoint of therapeutic success would be lower when applying the 2019 FDA guidance to this clinical trial, as compared to the proportion who achieved clinical resolution alone, as originally defined in the pragmatic clinical trial by Huttner et al.

## METHODS

### Study Design and Setting

We conducted a retrospective, patient-level reanalysis of the de-identified data from the intent-to-treat (ITT) population of the initial clinical trial. The reanalysis was performed according to a prespecified protocol based on the 2019 FDA uUTI guidance and the reanalysis was performed post hoc (ie, not before unblinding).

The reanalysis study population was derived from the investigator-initiated, multinational, open-label, analyst-blinded, randomized clinical trial of nitrofurantoin versus fosfomycin for the treatment of uUTI [8]. The study design of the clinical trial has been previously published [8]. In brief, nonpregnant women aged ≥18 years were included in the clinical trial if they had at least 1 symptom consistent with uUTI (dysuria, urgency, frequency, or suprapubic tenderness), a positive urine dipstick, and no known colonization or previous infection with uropathogens resistant to the study antibiotics. Recruitment of both ambulatory and hospitalized patients took place from October 2013 through April 2017 in Geneva, Switzerland; Lodz, Poland; and Petah-Tiqva, Israel.

Patients in the initial clinical trial were randomized on a 1:1 ratio to receive either nitrofurantoin 100 mg 3 times a day for 5 days or a single 3-g dose of fosfomycin. Women attended follow-up visits at days 14 (±2) and 28 (±7) post–treatment completion, during which clinical symptoms were evaluated and voided midstream urine specimens were collected and cultured.

### Inclusion in Reanalysis

The key methodological differences between the initial clinical trial and the requirements of the current FDA guidance are described in Table 1. In accordance with the current FDA guidance [5], women who participated in the initial clinical trial were eligible for inclusion in the reanalysis [modified ITT [mITT]) if they met all of the following criteria at baseline: 2 or more signs/symptoms of uUTI (dysuria, urinary frequency, urinary urgency, or suprapubic tenderness/pain) and identification of a single species of bacteria on pure culture at ≥10^5^ CFU/mL [5].

**Table 1.** Comparison of Key Methodological Differences Between the Initial Clinical Trial by Huttner et al (2018) and the 2019 Food and Drug Administration Guidance for Uncomplicated Urinary Tract Infection

Criteria/Endpoint	Huttner et al, 2018 [8]	FDA 2019 uUTI Guidance
Clinical inclusion criteria	≥1 symptom (dysuria, frequency, urgency, suprapubic tenderness^a^)	≥2 symptoms (dysuria, frequency, urgency, suprapubic pain^a^)
Microbiological inclusion criteria	Urine dipstick test result positive for either nitrites or leukocyte esterase	Single species of bacteria on pure culture at ≥10^5 ^CFU/mL
Primary endpoint	Clinical response at day 28 ± 7 post–treatment completion	Composite outcome of clinical and microbiological response at a fixed timepoint after randomization based on duration and half-life of the drug
Definition of primary endpoint	Clinical success defined as: Resolution of symptoms AND no need for additional antibiotics.	Clinical success defined as: Resolution of symptoms present at baseline AND no new symptoms. Microbiological success defined as: Reduction in infecting bacterial pathogen to <10^3^ CFU/mL.
Definition of secondary endpoint	Microbiological resolution defined as: Eradication of the infecting bacterial strain with no recurrence of bacteriuria (ie, <10^3^ CFU/mL). Microbiological failure defined as: Bacteriuria ≥10^3 ^CFU/mL with the infecting bacterial strain.	…
Handling of missing primary endpoint data	Patients with missing data were excluded from the primary analysis (unless there was documented clinical failure prior to the primary endpoint).	Patients should not be excluded from the microbiological intent-to-treat population due to events occurring after randomization (eg, loss to follow-up). As a result, patients with missing clinical and/or microbiological response data are unable to demonstrate therapeutic success and should therefore be considered as therapeutic failure.

Abbreviations: CFU, colony-forming units; FDA, United States Food and Drug Administration; uUTI, uncomplicated urinary tract infection.

^a^For pragmatic purposes, suprapubic pain and tenderness were treated as equivalent in the reanalysis.

### Outcome Measures

As recommended by the current FDA guidance, the primary endpoint—therapeutic response—was a composite outcome of clinical and microbiological responses. Clinical resolution required complete resolution of the uUTI signs and symptoms present at trial entry, no new signs/symptoms, or use of anonstudy antibiotic for the treatment of uUTI. Microbiological success was defined as a reduction in the causative pathogen of the initial uUTI episode to <10^3^ CFU/mL. The composite primary outcome—therapeutic success—therefore required the criteria for clinical resolution and microbiological success to have been met and was evaluated at the predefined visits at days 14 and 28 post–treatment completion. Where follow-up data were missing, such that it was not possible to determine clinical or microbiological responses during the follow-up period, clinical or microbiological outcomes were considered indeterminate. For the primary efficacy endpoint, patients with indeterminate clinical or microbiological responses were not excluded from the reanalysis and were conservatively treated as therapeutic failure.

In the initial clinical trial, “indeterminate” was defined as either persistence of symptoms without objective evidence of infection or any extenuating circumstances precluding a classification of clinical resolution or failure, and “missing” was defined as patients with missing data for clinical response without prior failure and who were excluded from the primary analysis [8]. In the reanalysis, “indeterminate” was defined as missing data at the day 14 or day 28 post–treatment completion visit and these patients were included in the analyses.

### Statistical Analyses

The demographic, clinical, and microbiological characteristics of women in ITT population of the initial clinical trial were compared to those women eligible for inclusion in the reanalysis mITT population. We expressed continuous variables as the mean and standard deviation or as the median and interquartile range if their distribution was skewed, and categorical variables were expressed as counts and percentages. Because of the explorative nature of this secondary analysis, hypothesis testing for statistical significance was not conducted.

Consistent with the current FDA guidance [5], the primary composite endpoint among women eligible for inclusion in the reanalysis was calculated and then compared to the proportion of women who met the criteria for clinical resolution alone, as originally defined in the initial clinical trial, at days 14 and 28 post–treatment completion.

In sensitivity analyses, the impact of the culture threshold of the infecting strain at baseline was evaluated. This was achieved by expanding the microbiological eligibility criteria at baseline for inclusion in the reanalysis mITT to the identification of a single species of bacteria on pure culture at ≥10^4^ CFU/mL, and then to the identification of a single species of bacteria on pure culture at ≥10^3^ CFU/mL. Additional sensitivity analyses were performed by restricting the eligibility criteria at baseline for inclusion in the reanalysis to those patients with a single species of *Escherichia coli* identified on pure culture at ≥10^5^ CFU/mL.

All analyses were performed using R, version 4.0.0 software (http://cran.r-project.org).

### Patient Consent Statement

The initial clinical trial was approved by the independent ethics committees of each site and by the Swiss Agency for Therapeutic Products (2013DR4095). The trial was conducted in accordance with the ethical principles stated in the most recent version of the Declaration of Helsinki, the International Council for Harmonisation of Technical Requirements for Pharmaceuticals for Human Use (ICH) Guideline for Good Clinical Practice (E6), the Consolidated Standards of Reporting Trials (CONSORT) statement, and the relevant Swiss legislation (LPTh, OClin) and is registered on ClinicalTrials.gov under the identifier NCT01966653. All participants in the initial clinical trial provided written informed consent before their inclusion.

## RESULTS

**Figure 1.**
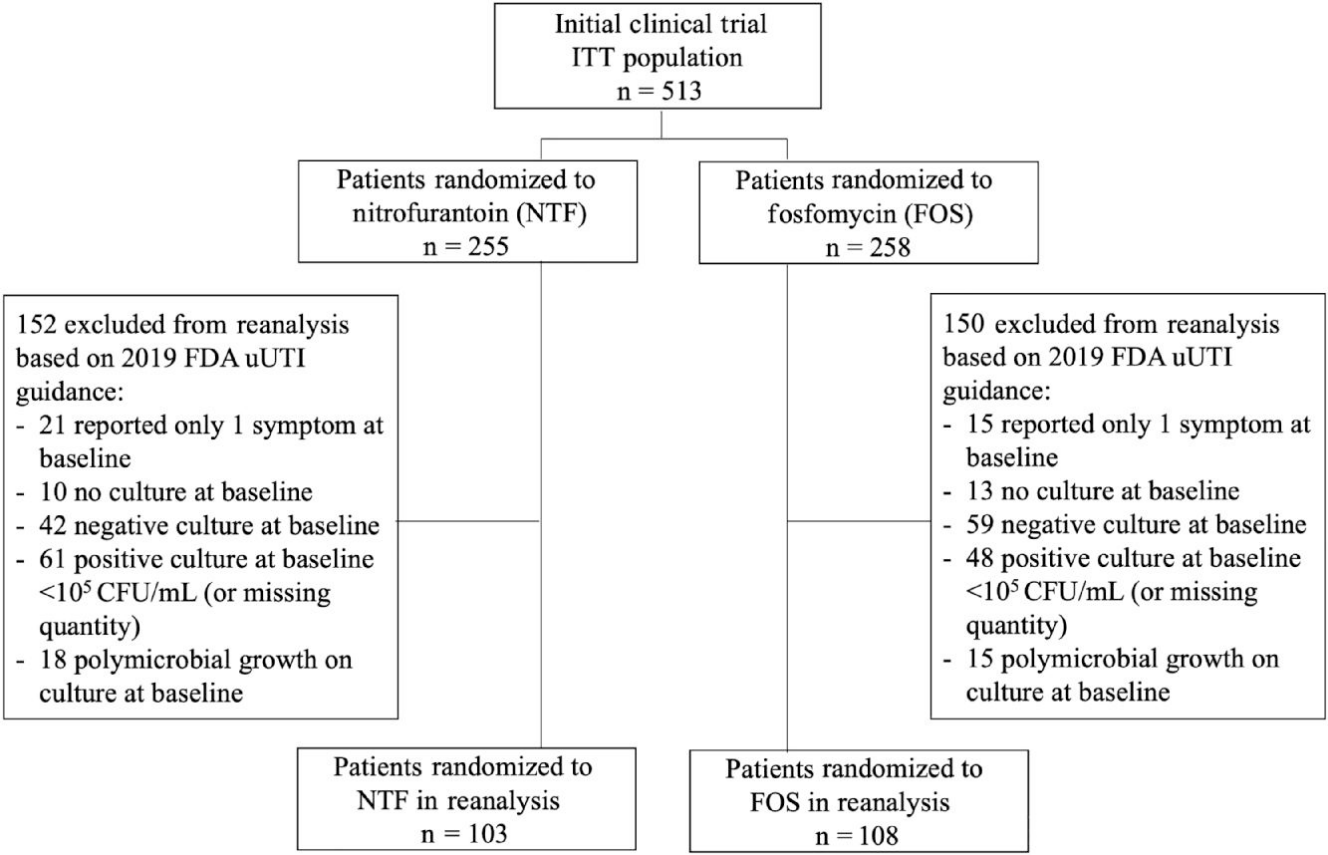
Inclusion of patients from the initial clinical trial in the reanalysis modified intent-to-treat population. Abbreviations: CFU, colony-forming units; FDA, United States Food and Drug Administration; FOS, fosfomycin; ITT, intent-to-treat; NTF, nitrofurantoin; uUTI, uncomplicated urinary tract infection.

Between October 2013 through April 2017, 513 nonpregnant women aged ≥18 years participated in the initial clinical trial; 255 received nitrofurantoin and 258 received fosfomycin (ITT population). Of these 513 women, 211 (41%) met the inclusion criteria as recommended in the current FDA guidance and were included in the reanalysis mITT population: 103 patients in the nitrofurantoin arm and 108 patients in the fosfomycin arm (Figure 1).

Table 2 shows the demographic, clinical, and microbiological characteristics of the initial ITT study population and the reanalysis mITT population. The demographic, clinical, and microbiological characteristics between the 2 intervention arms remained relatively well balanced and comparable to the initial ITT clinical trial population. The only exception was the age of the patients retained in the fosfomycin arm (median, 56 years), which was higher than that of patients in the nitrofurantoin arm (median, 43 years), and higher than patients in the fosfomycin arm in the initial ITT clinical trial (median, 46 years). The predominant uropathogen in the reanalysis mITT population was *Escherichia coli*, followed by *Klebsiella* spp and *Proteus* spp.

**Table 2.** Baseline Demographic, Clinical, and Microbiological Characteristics of the Initial Intent-to-Treat Clinical Trial Population and Reanalysis Modified Intent-to-Treat Population

Characteristic	Initial ITT Clinical Trial Population				Reanalysis mITT Population			
	Nitrofurantoin		Fosfomycin		Nitrofurantoin		Fosfomycin	
	No. (n = 255)	(%)	No. (n = 258)	(%)	No. (n = 103)	(%)	No. (n = 108)	(%)
Study site								
Geneva, Switzerland	94/255	(37)	92/258	(36)	39/103	(38)	40/108	(37)
Lodz, Poland	98/255	(38)	102/258	(40)	48/103	(47)	50/108	(46)
Petah-Tiqva, Israel	63/255	(25)	64/258	(24)	16/103	(15)	18/108	(17)
Age, y								
Median (IQR)	43	(31–63)	46	(31–66)	43	(29–62)	56	(34–71)
18–50 y	148/255	(58)	143/258	(55)	56/103	(54)	45/108	(42)
>50 y	106/255	(42)	115/258	(45)	47/103	(46)	63/108	(58)
Initial urinary symptomatology								
Dysuria	198/255	(78)	196/258	(75)	88/103	(85)	87/108	(81)
Urgency	195/255	(77)	204/258	(79)	86/103	(83)	87/108	(81)
Frequency	222/255	(87)	224/258	(87)	96/103	(93)	99/108	(92)
Suprapubic pain/tenderness	134/255	(52)	131/258	(51)	52/103	(50)	52/108	(48)
Antibiotic therapy for any reason in the past year	131/255	(52)	137/258	(53)	62/103	(60)	61/108	(56)
Any previous UTI	41/255	(16)	43/258	(17)	17/103	(16)	22/108	(20)
Baseline urinalysis results								
Positive for nitrites only	7/255	(3)	10/258	(4)	2/103	(2)	4/108	(4)
Positive for leukocytes only	131/255	(52)	152/258	(59)	36/103	(35)	46/108	(43)
Positive for both nitrites and leukocytes	115/255	(45)	94/258	(37)	65/103	(63)	58/108	(54)
Uropathogen detected in urine culture at baseline:								
*Escherichia coli*	111/194	(57)	119/184	(65)	75/103	(73)	85/108	(79)
*Klebsiella* spp	20/194	(10)	7/184	(4)	15/103	(14)	6/108	(5)
*Proteus* spp	7/194	(4)	10/184	(5)	3/103	(3)	5/108	(5)
*Enterococcus* spp	13/194	(7)	14/184	(8)	2/103	(2)	2/108	(2)
Group B *Streptococcus*	7/194	(4)	6/184	(3)	2/103	(2)	4/108	(4)
*Enterobacter* spp	5/194	(3)	4/184	(2)	2/103	(2)	1/108	(1)
Mixed flora	51/194	(26)	40/184	(21)	0	(0)	0	(0)
Other	10/194	(5)	7/184	(4)	4/103	(4)	5/108	(5)

Abbreviations: IQR, interquartile range; ITT, intent-to-treat; mITT, modified intent-to-treat; UTI, urinary tract infection.

### Outcome Measures

**Figure 2.**
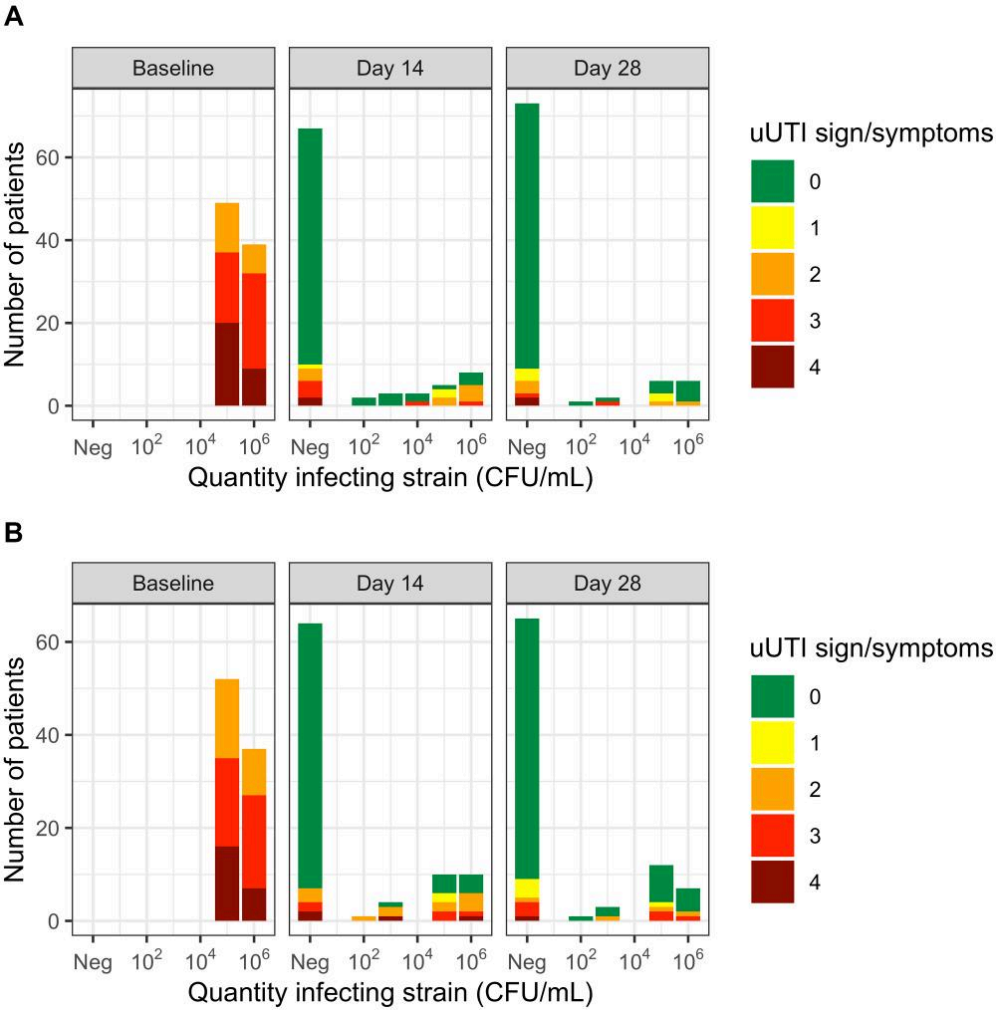
Quantity of infecting strain by number of signs/symptoms at baseline and day 14 and day 28 post–treatment completion by intervention arm (nitrofurantoin [*A*] versus fosfomycin [*B*]) in patients in the reanalysis modified intent-to-treat study population with complete clinical and microbiological data at follow-up visits (nitrofurantoin, n = 88; fosfomycin, n = 89). Abbreviations: CFU, colony-forming units; Neg, negative culture; uUTI, uncomplicated urinary tract infection.

Table 3 shows the primary and secondary outcomes as defined in the initial clinical trial and in the reanalysis mITT population in accordance with the current FDA guidance. The proportions of patients who met the criteria for clinical success at days 14 and 28 post–treatment completion were similar between the initial clinical trial and the reanalysis mITT population (Table 3, Supplementary Figure 1). The proportions of patients who met the criteria for microbiological success at day 14 post–treatment completion was lower in the reanalysis mITT population, particularly in the nitrofurantoin group, as compared to the initial clinical trial (Table 3). The proportion of patients who met the criteria for microbiological success at day 28 was similar in the reanalysis mITT population as compared to the initial clinical trial (Table 3). Figure 2 shows the extent to which the clinical responses aligned with microbiological responses over the course of the follow-up period among those patients who had complete clinical and microbiological outcome data at both follow-up visits. With respect to the primary composite efficacy endpoint, 59% (61/103) of patients in the nitrofurantoin group and 57% (62/108) of patients in the fosfomycin group achieved therapeutic success at day 14 post–treatment completion. The composite efficacy rates at day 28 post–treatment completion were, as might be expected, slightly lower in both groups, as compared to the corresponding efficacy rates at day 14 post–treatment completion (Table 3).

**Table 3.** Comparison of Primary and Secondary Outcomes at Days 14 and 28 Post–Treatment Completion Between the Initial Clinical Trial Intent-to-Treat Study Population and Reanalysis Modified Intent-to-Treat Population

Outcome	Initial Clinical Trial ITT				Reanalysis mITT			
	Nitrofurantoin		Fosfomycin		Nitrofurantoin		Fosfomycin	
	No. (n = 255)	(%)	No. (n = 258)	(%)	No. (n = 103)	(%)	No. (n = 108)	(%)
Primary outcomes								
Clinical response at day 14								
Clinical resolution	184/247	(75)	162/247	(66)	76/103	(74)	75/108	(69)
Clinical failure	56/247	(23)	75/247	(30)	23/103	(22)	30/108	(28)
Indeterminate^a^	7/247	(3)	10/247	(4)	4/103	(4)	3/108	(3)
Missing	8	…	11	…	…	…	…	…
Microbiological response at day 14								
Microbiological success	146/177	(82)	121/165	(73)	72/103	(70)	72/108	(67)
Microbiological failure	31/177	(18)	44/165	(27)	19/103	(18)	27/108	(25)
Indeterminate^a^	…	…	…	…	12/103	(12)	9/108	(8)
Therapeutic response at day 14								
Therapeutic success	…	…	…	…	61/103	(59)	62/108	(57)
Therapeutic failure	…	…	…	…	42/103	(41)	46/108	(43)
Secondary outcomes								
Clinical response at day 28								
Clinical resolution	171/244	(70)	139/241	(58)	67/103	(65)	64/108	(59)
Clinical failure	66/244	(27)	94/241	(39)	29/103	(28)	35/108	(32)
Indeterminate^a^	7/244	(3)	8/241	(3)	7/103	(7)	9/108	(8)
Missing	11	…	17	…	…	…	…	…
Microbiological response at day 28								
Microbiological success	129/175	(74)	103/163	(63)	76/103	(74)	70/108	(65)
Microbiological failure	46/175	(26)	60/163	(37)	16/103	(16)	23/108	(21)
Indeterminate^a^	…	…	…	…	11/103	(11)	15/108	(14)
Therapeutic response at day 28								
Therapeutic success	…	…	…	…	56/103	(54)	53/108	(49)
Therapeutic failure	…	…	…	…	47/103	(46)	55/108	(51)

Abbreviations: ITT, intent-to-treat; mITT, modified intent-to-treat.

^a^In the initial clinical trial, “indeterminate” was defined as either persistence of symptoms without objective evidence of infection or any extenuating circumstances precluding a classification of clinical resolution or failure, and “missing” was defined as patients with missing data for clinical response without prior failure and who were excluded from the primary analysis. In the reanalysis, “indeterminate” was defined as missing data at the day 14 or day 28 post–treatment completion visit.

### Sensitivity Analyses

In sensitivity analyses, expanding the microbiological criteria at baseline to ≥10^4^ CFU/mL and ≥10^3^ CFU/mL on demographic resulted in study populations with similar clinical and microbiological characteristics (Supplementary Table 1). In addition, the proportion of therapeutic success at day 14 post–treatment completion in these populations was similar when the microbiological criteria at baseline were expanded to include patients from the initial clinical trial with a single species of bacteria on pure culture at ≥10^4^ CFU/mL, and then to patients with a single species of bacteria on pure culture at ≥10^3^ CFU/mL, as compared to the microbiological criteria at baseline used in the primary reanalysis (Supplementary Table 2).

In additional sensitivity analyses, the proportion of therapeutic success at day 14 post–treatment completion was similar when restricting the eligibility criteria at baseline for inclusion in the reanalysis to those patients with a single species of *Escherichia coli* identified on pure culture at ≥10^5^ CFU/mL (Supplementary Table 3).

## DISCUSSION

In this retrospective, patient-level, post hoc protocol-based reanalysis of the data from the clinical trial of nitrofurantoin versus fosfomycin for the treatment of uUTI, we applied the eligibility criteria and endpoint definitions in accordance with the 2019 FDA uUTI guidance. Among the patients who had participated in the initial clinical trial and who were eligible for inclusion in the reanalysis, 59% of patients in the nitrofurantoin group and 57% of fosfomycin-treated patients, respectively, met the composite therapeutic success endpoint recommended by the FDA at the predefined day 14 post–treatment completion visit. This was lower than the 75% of patients in the nitrofurantoin group and 66% of patients in the fosfomycin group who achieved complete clinical resolution at day 14 post–treatment completion in the initial clinical trial [8]. It was also lower than the 70% of patients in the nitrofurantoin group and 58% of patients in the fosfomycin group who achieved the primary endpoint in the initial clinical trial, defined as complete clinical resolution at day 28 post–treatment completion [8].

The application of the current FDA guidance on the retrospective, patient-level reanalysis of the clinical trial had 2 main impacts. First, as the microbiological criteria at baseline required a single species of bacteria on pure culture at ≥10^5^ CFU/mL, almost 60% of the initial clinical trial's ITT population was not eligible for inclusion in the reanalysis. This requirement increases the number of patients who must be enrolled to yield the required number of microbiologically evaluable subjects (ie, trial burden). The introduction of microbiological components in the inclusion and primary endpoint criteria, as required by the FDA [5] and EMA [7], is important as these criteria provide objective, measurable, reproducible, and verifiable data that are needed in registrational trials. Nonetheless, the ≥10^5^ CFU/mL threshold at baseline appears to arbitrarily exclude infections based on clinical presentation alone. In sensitivity analyses, we were able to show clinical uUTI caused by uropathogens at 10^4^ CFU/mL and 10^3^ CFU/mL. Beyond this study, clinical uUTI caused by *Escherichia coli* have been demonstrated at quantities as low as 10^2^ CFU/mL [9].

For future clinical trials that will need to adhere to the current FDA and EMA recommendations, the ≥10^5^ CFU/mL threshold carries important cost and logistic considerations for clinical trialists as the stringency of the inclusion criteria are such that many more patients will need to be screened in order to have a sufficient number of patients with a uropathogen (single species of bacteria on pure culture recommended) at ≥10^5^ CFU/mL.

Second, as the primary composite endpoint requires that the criteria for both clinical and microbiological success be met, lower composite efficacy rates were observed when current FDA guidance was applied. When considering clinical response alone, 74% (76/103) and 69% (75/108) of patients in the nitrofurantoin and fosfomycin groups in the reanalysis mITT, respectively, achieved clinical resolution by day 14 post–treatment completion. These results were similar to those achieved in the initial clinical trial, in which 75% (184/247) and 66% (162/247) of patients in the nitrofurantoin and fosfomycin groups, respectively, achieved clinical resolution at day 14 post–treatment completion. For uUTI, clinical resolution is most meaningful to patients and these infections are most commonly treated empirically. For complicated UTI, microbiological failure has been associated with increased risk for clinical relapse [10]. It is unclear the extent to which this may also be the case for uUTI, and the follow-up period of the initial trial does not allow us to explore this. However, obtaining post–treatment cultures to document the decline in growth of the infecting pathogen is inconsistent with current treatment recommendations [1] and clinical practice [11, 12]. Importantly, the treatment of persistent bacteriuria without accompanying symptoms has been identified as an important contributor to inappropriate antimicrobial use, which promotes resistance [13, 14].

Our findings have additional implications for future registrational and nonregistrational clinical trials. The difference in efficacy between nitrofurantoin and fosfomycin that was originally observed in the initial clinical trial was markedly reduced when comparing the clinical response observed in the initial trial to the composite therapeutic response in the reanalysis. This may be an important consideration for future trials that use nitrofurantoin as a comparator, as this may limit the ability to compare treatment effects of these antibiotics in historical trials with those of future clinical trials. This finding is also in contrast to the evidence that supports its use as one of the recommended first-line therapeutics for uUTI [15, 16].

Our study nonetheless has several limitations. First, the initial clinical trial was a pragmatic, real-world, investigator-initiated clinical trial of 2 first-line therapeutics for uUTI. That is, the conditions were those of a phase 4 trial, rather than a registrational trial. Missing data at baseline excluded patients from inclusion in the reanalysis and missing data at follow-up were conservatively treated as therapeutic failure. This may have resulted in an overestimation of the proportion of therapeutic failures, as compared to the real-life treatment effect. Second, the initial clinical trial was conducted across 3 study sites and the laboratory analyses were not centralized, potentially leading to heterogeneity in microbiological methods across study sites.

Overall, in a reanalysis of a randomized clinical trial of women with uncomplicated UTI, the application of current FDA guidance resulted in a lower efficacy rate based on composite therapeutic success rates versus clinical resolution alone observed in the initial clinical trial. These criteria may limit the ability to compare antibiotic treatment effects between historical and future clinical trials adhering to current regulatory guidance.

## Supplementary Material

ofad557_Supplementary_Data

## Supplementary Data

Supplementary materials are available at *Open Forum Infectious Diseases* online. Consisting of data provided by the authors to benefit the reader, the posted materials are not copyedited and are the sole responsibility of the authors, so questions or comments should be addressed to the corresponding author.

## Notes

***Author contributions.*** M. G.-C., L. L., A. H., and S. H. designed and obtained funding for the initial clinical trial. M. G.-C., L. L., A. H., and S. H. oversaw the collection and analysis of data from the initial clinical trial. W. F., C. H., D. F., S. J., A. M., and S. H. developed the protocol for the reanalysis based on the 2019 FDA uUTI guidance. R. G. and A. B. performed the statistical analyses. R. G., A. B., and S. H. drafted the first version of the manuscript. All authors critically reviewed and approved the final version of the manuscript.

***Acknowledgments.*** The authors thank all trial participants for their participation in the initial clinical trial, as well as the staff who were involved in the implementation of the trial in each of the 3 study sites: Geneva, Switzerland; Lodz, Poland; and Petah-Tiqva, Israel.

The authors are grateful for feedback on the draft protocol and interpretation of the results from Caroline Perry, Amanda Sheets, Jeremy Dennison, Helen Millns, Nicole Scangarella-Oman, Fanny Mitrani-Gold, and Madison Preib, as well as the administrative assistance provided by Grace Richmond and Shilpa Patkar, all of whom are employed by GSK.

***Data availability.*** The data from this study and the initial clinical trial are not publicly available.

***Financial support.*** The initial clinical trial was funded by the European Commission under the Life Science Health Priority of the 7th Framework Program (Assessment of Clinical Efficacy by a Pharmacokinetic/Pharmacodynamic Approach to Optimize Effectiveness and Reduce Resistance for Off-Patent Antibiotics project, grant agreement 278348). The Lodz site received additional funding from the Polish Ministry of Science and Higher Education (2014–2017, contract 2979/7.PR/2014/2). This patient-level reanalysis of the initial clinical trial was funded by GSK (study 219539). GSK developed the protocol for the reanalysis, in consultation with the Geneva University Hospital and Faculty of Medicine at the University of Geneva, Switzerland. However, all data analyses were conducted by the Geneva University Hospital and Faculty of Medicine at the University of Geneva, Switzerland, and the data were not transferred to GSK.
