# Supplementary material for: Impact of the 2019 Food and Drug Administration Guidance for Uncomplicated Urinary Tract Infection on Treatment Response Rates: A Reanalysis of a Clinical Trial of Nitrofurantoin vs Fosfomycin
Source: Open Forum Infect Dis. 2023 Nov 4;10(11):ofad557. doi: 10.1093/ofid/ofad557 (PMC10661658; doi:10.1093/ofid/ofad557)
Supplement: ofad557_Supplementary_Data [file ofad557_supplementary_data.pdf]

Impact of the 2019 FDA Guidance for Uncomplicated Urinary Tract Infection on Treatment Response Rates: a Re-analysis of a Clinical Trial of Nitrofurantoin vs Fosfomycin

Supplementary Material

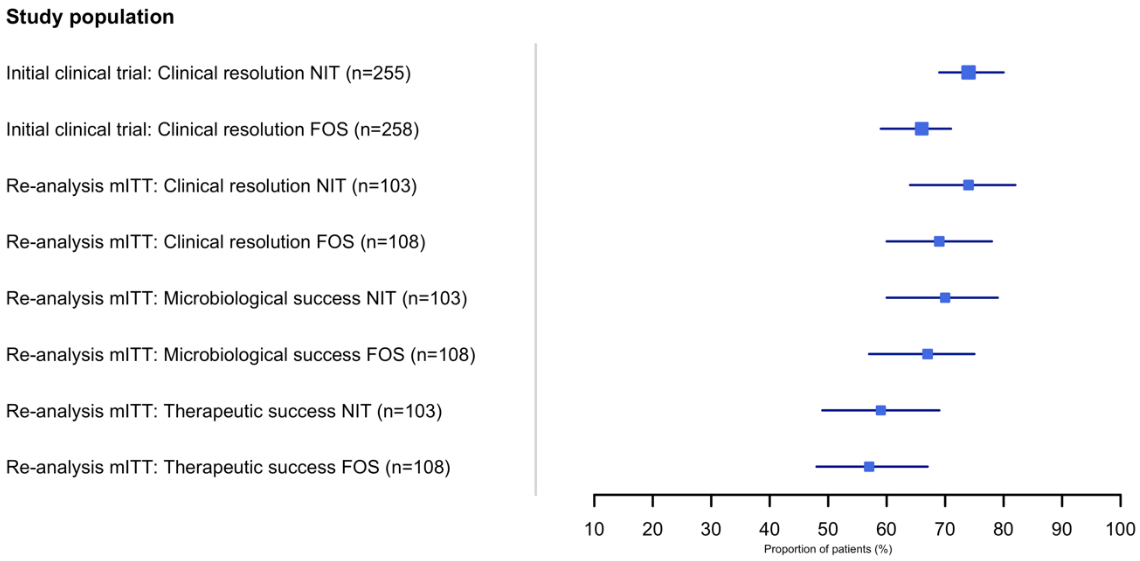

Supplementary Figure 1. Comparison of outcomes (95% CI) at day 14 post-treatment completion between the initial clinical trial ITT study population and the re-analysis mITT study population (NIT: Nitrofurantoin; FOS: Fosfomycin)

Supplementary Table 1. Impact of the culture threshold of the infecting strain at baseline on demographic, clinical and microbiological characteristics of re-analysis mITT populations

	**Re-analysis mITT**				**Re-analysis mITT-10^{4} ***				**Re-analysis mITT-10^{3} +**			
	Nitrofurantoin		Fosfomycin		Nitrofurantoin		Fosfomycin		Nitrofurantoin		Fosfomycin	
	n=103	(%)	n=108	(%)	n=113	(%)	n=121	(%)	n=117	(%)	n=130	(%)
Study site:												
- Geneva, Switzerland	39/103	(38)	40/108	(37)	46/113	(41)	48/121	(40)	49/117	(42)	52/130	(40)
- Lodz, Poland	48/103	(47)	50/108	(46)	50/113	(44)	54/121	(45)	51/117	(44)	59/130	(45)
- Petah-Tiqva, Israel	16/103	(15)	18/108	(17)	17/113	(15)	19/121	(16)	17/117	(15)	19/130	(15)
Age:												
- median (IQR)	43	(29-62)	56	(34-71)	43	(30-62)	54	(34-70)	44	(31-63)	52	(32-68)
- 18-50 years	56/103	(54)	45/108	(42)	63/113	(56)	55/121	(45)	64/117	(55)	63/130	(48)
- >50 years	47/103	(46)	63/108	(58)	50/113	(44)	66/121	(55)	53/117	(45)	67/130	(52)
Initial urinary symptomatology, No. (%):												
- dysuria	88/103	(85)	87/108	(81)	96/113	(85)	96/121	(79)	99/117	(85)	103/130	(79)
- urgency	86/103	(83)	87/108	(81)	95/113	(84)	100/121	(83)	98/117	(84)	109/130	(84)
- frequency	96/103	(93)	99/108	(92)	106/113	(94)	111/121	(92)	109/117	(93)	120/130	(92)
- suprapubic pain/tenderness	52/103	(50)	52/108	(48)	57/113	(50)	60/121	(50)	59/117	(50)	65/130	(50)
Antibiotic therapy for any reason in the past year, No. (%)	62/103	(60)	61/108	(56)	66/113	(58)	70/121	(58)	69/117	(59)	78/130	(60)
Any previous UTI, No. (%)	17/103	(16)	22/108	(20)	19/113	(17)	25/121	(21)	19/117	(16)	26/130	(20)
Baseline urinalysis results, No. (%):												
- positive for nitrites only	2/103	(2)	4/108	(4)	2/113	(2)	4/121	(3)	3/117	(2)	5/130	(4)
- positive for leukocytes only	36/103	(35)	46/108	(43)	43/113	(38)	53/121	(44)	44/117	(38)	60/130	(46)
- positive for both nitrites and leukocytes	65/103	(63)	58/108	(54)	68/113	(60)	64/121	(53)	70/117	(60)	65/130	(50)
Uropathogen detected in urine culture at baseline:												
- *Escherichia coli*	75/103	(73)	85/108	(79)	80/113	(71)	94/121	(78)	81/117	(69)	97/130	(75)
- *Klebsiella* spp	15/103	(14)	6/108	(5)	16/113	(14)	6/121	(5)	16/117	(14)	6/130	(5)
- *Proteus* spp	3/103	(3)	5/108	(5)	3/113	(3)	5/121	(4)	3/117	(2)	5/130	(4)
- *Enterococcus* spp	2/103	(2)	2/108	(2)	2/113	(2)	3/121	(2)	3/117	(2)	5/130	(4)
- Group B *Streptococcus*	2/103	(2)	4/108	(4)	3/113	(3)	4/121	(3)	3/117	(2)	5/130	(4)
- *Enterobacter* spp	2/103	(2)	1/108	(1)	2/113	(2)	1/121	(1)	2/117	(2)	1/130	(1)
- Other	4/103	(4)	5/108	(5)	7/113	(6)	8/121	(7)	9/117	(8)	11/130	(8)

* Eligibility criteria for inclusion: women who participated in the initial clinical trial with two or more signs/symptoms of uUTI (dysuria, urinary frequency, urinary urgency or suprapubic tenderness/pain); and identification of a single species of bacteria on pure culture at $\geq 10^4$ CFU/mL.
+ Eligibility criteria for inclusion: women who participated in the initial clinical trial with two or more signs/symptoms of uUTI (dysuria, urinary frequency, urinary urgency or suprapubic tenderness/pain); and identification of a single species of bacteria on pure culture at $\geq 10^3$ CFU/mL.

Supplementary Table 2. Impact of the culture threshold of the infecting strain at baseline on primary outcomes at day 14 post-treatment completion

	**Re-analysis mITT**				**Re-analysis mITT-10^{4} ***				**Re-analysis mITT-10^{3} +**			
	Nitrofurantoin		Fosfomycin		Nitrofurantoin		Fosfomycin		Nitrofurantoin		Fosfomycin	
	n=103	(%)	n=108	(%)	n=113	(%)	n=121	(%)	n=117	(%)	n=130	(%)
Primary outcomes												
Clinical response at day 14												
- Clinical resolution	76/103	(74)	75/108	(69)	84/113	(74)	82/121	(68)	87/117	(74)	90/130	(69)
- Clinical failure	23/103	(22)	30/108	(28)	25/113	(22)	35/121	(29)	26/117	(22)	36/130	(28)
- Indeterminate	4/103	(4)	3/108	(3)	4/113	(4)	4/121	(3)	4/117	(3)	4/130	(3)
Microbiological response at day 14												
- Microbiological success	72/103	(70)	72/108	(67)	80/113	(71)	82/121	(68)	83/117	(71)	87/130	(67)
- Microbiological failure	19/103	(18)	27/108	(25)	20/113	(18)	29/121	(24)	21/117	(18)	32/130	(25)
- Indeterminate	12/103	(12)	9/108	(8)	13/113	(12)	10/121	(8)	13/117	(11)	11/130	(8)
Therapeutic response at day 14												
- Therapeutic success	61/103	(59)	62/108	(57)	67/113	(59)	69/121	(57)	70/117	(60)	73/130	(56)
- Therapeutic failure	42/103	(41)	46/108	(43)	46/113	(41)	52/121	(43)	47/117	(40)	57/130	(44)

* Eligibility criteria for inclusion: women who participated in the initial clinical trial with two or more signs/symptoms of uUTI (dysuria, urinary frequency, urinary urgency or suprapubic tenderness/pain); and identification of a single species of bacteria on pure culture at ≥10^4 CFU/mL.

+ Eligibility criteria for inclusion: women who participated in the initial clinical trial with two or more signs/symptoms of uUTI (dysuria, urinary frequency, urinary urgency or suprapubic tenderness/pain); and identification of a single species of bacteria on pure culture at ≥10^3 CFU/mL.

Supplementary Table 3. Comparison of primary and secondary outcomes at days 14 and 28 post treatment completion between the re-analysis mITT population and those patients with a single species of *Escherichia coli* identified on pure culture at $\geq 10^5$ CFU/mL

	**Re-analysis mITT**				**Re-analysis mITT *E. coli****			
	Nitrofurantoin		Fosfomycin		Nitrofurantoin		Fosfomycin	
	n=103	(%)	n=108	(%)	n=75	(%)	n=85	(%)
Primary outcomes								
Clinical response at day 14								
- Clinical resolution	76/103	(74)	75/108	(69)	54/75	(72)	61/85	(72)
- Clinical failure	23/103	(22)	30/108	(28)	17/75	(23)	21/85	(25)
- Indeterminate	4/103	(4)	3/108	(3)	4/75	(5)	3/85	(4)
Microbiological response at day 14								
- Microbiological success	72/103	(70)	72/108	(67)	52/75	(69)	58/85	(68)
- Microbiological failure	19/103	(18)	27/108	(25)	12/75	(16)	21/85	(25)
- Indeterminate	12/103	(12)	9/108	(8)	11/75	(15)	6/85	(7)
Therapeutic response at day 14								
- Therapeutic success	61/103	(59)	62/108	(57)	44/75	(59)	53/85	(62)
- Therapeutic failure	42/103	(41)	46/108	(43)	31/75	(41)	32/85	(38)
Secondary outcomes								
Clinical response at day 28								
- Clinical resolution	67/103	(65)	64/108	(59)	47/75	(63)	52/85	(61)
- Clinical failure	29/103	(28)	35/108	(32)	21/75	(28)	24/85	(28)
- Indeterminate	7/103	(7)	9/108	(8)	7/75	(9)	9/85	(11)
Microbiological response at day 28								
- Microbiological success	76/103	(74)	70/108	(65)	53/75	(71)	55/85	(65)
- Microbiological failure	16/103	(16)	23/108	(21)	12/75	(16)	19/85	(22)
- Indeterminate	11/103	(11)	15/108	(14)	10/75	(13)	11/85	(13)
Therapeutic response at day 28								
- Therapeutic success	56/103	(54)	53/108	(49)	41/75	(55)	43/85	(51)
- Therapeutic failure	47/103	(46)	55/108	(51)	34/75	(45)	42/85	(49)

[*] Eligibility criteria for inclusion: women who participated in the initial clinical trial with two or more signs/symptoms of uUTI (dysuria, urinary frequency, urinary urgency or suprapubic tenderness/pain); and identification of a single species of *Escherichia coli* on pure culture at $\geq 10^5$ CFU/mL
